# High-Concentrate Feeding to Dairy Cows Induces Apoptosis via the NOD1/Caspase-8 Pathway in Mammary Epithelial Cells

**DOI:** 10.3390/genes11010107

**Published:** 2020-01-17

**Authors:** Zain ul Aabdin, Xiaoye Cheng, Hongyu Dai, Yan Wang, Benazir Sahito, Animesh Chandra Roy, Meena Arif Memon, Xiangzhen Shen

**Affiliations:** College of Veterinary Medicine, Nanjing Agricultural University, Nanjing 210095, China; drzainsahito@hotmail.com (Z.u.A.); 2017107103@njau.edu.cn (X.C.); 2016207036@njau.edu.cn (H.D.); 2018207030@njau.edu.cn (Y.W.); DRbena22@hotmail.com (B.S.); royanimeshvet98@yahoo.com (A.C.R.); vetpet76@yahoo.com (M.A.M.)

**Keywords:** mammary epithelial cell apoptosis, iE-DAP, NOD1, SARA, dairy cows

## Abstract

(1) Background: The effects of a high-concentrate (HC) diet in inducing mammary epithelial cell apoptosis in dairy cows via the NOD1/Caspase-8 pathway have never been investigated before the current study. (2) Methods: Twelve Holstein Frisian cows at mid-lactation were selected to conduct this research. The animals were randomly allocated to two groups (n = 6), and both groups received one of two diets: a low-concentrate (LC) (forage: concentrate 6:4) or a high-concentrate (HC) (forage: concentrate 4:6) diet. Furthermore, an enzyme activity assay, tunnel cell assay, RT-qPCR, western blotting, and an immunofluorescence antibody (IFA) assay were performed to elucidate the effect of an HC diet in the mammary gland of dairy cows. (3) Results: The tunnel cell assay revealed a significant number of apoptotic cells in HC group, and the concentration of Caspase-3, and Caspase-8 was higher in the HC group than in the LC group. NOD1, Rip-2, Caspase-3, Caspase-8, Caspase-9, and Bax mRNA expressions, and NOD1, Caspase-3, Caspase-8, and Bax protein expressions, in the HC group were markedly higher than those in the LC group. Furthermore, Bcl-2 mRNA and protein expressions were markedly decreased in the HC compared to those in the LC group. (4) Conclusions: A HC diet fed to dairy cows incites subacute ruminal acidosis (SARA), which increases the iE-DAP concentration and induces apoptosis in the mammary gland via the NOD1/Caspase-8 pathway.

## 1. Introduction

Dairy ruminants are regularly nourished with high-grain forage to produce a higher milk yield in dairy farming industries. However, in ruminants, this high-grain forage is associated with subacute ruminal acidosis (SARA). When the rumen pH increases up to 5.5–5.8 for more than 180 minutes every day, it indicates that SARA is effectively incited [1]. When dairy animals are nourished with a composition of total mixed ration containing 20% mashed wheat, then SARA ought to be effectively prompted [2]. Laminitis, gastrointestinal damage, and liver abscesses are the clinical signs of SARA [3]. A decline in rumen pH leads to the lysis of rumen microorganisms and enhances the permeation ability of the rumen wall, which may allow substantial bacterial components to translocate into the blood circulation [4]. Many micro molecules, such as lipopolysaccharides (LPS) and D-glutamyl-meso-diaminopimelic acid (iE-DAP), are well-known to induce mammary cell inflammation [5]. Despite this, the facts regarding how iE-DAP induces cellular apoptosis in the mammary gland tissue of dairy cows because of an HC diet are not well-established.

The first line of defense against non-specific infections in phagocytes, mainly dendritic cells and macrophages, is termed innate immunity. The expression of pattern recognition receptors (PRRs) is a significant aspect of these cells, exhibiting extreme involvement in detecting pathogen-associated molecular patterns (PAMPs) [6,7]. The latest advances in the field of PRR have improved our knowledge in determining the role of the NOD-like receptor (NLR) family in sensing the infections caused by microorganisms [8]. NLRs are mostly expressed in epithelial cells, such as phagocytes, macrophages, and neutrophils, which can also be expressed by individual members of the NLR family. Distinct from Toll-like receptors (TLRs), NLRs are found in the cell cytosol [9]. NOD1 is a unique member of the NLR family and is universally expressed in many cells [7]. Peptidoglycan (PGN) is a preserved erection of Gram-negative microbes detected by NOD1 to sense bacterial infections [10]. However, the caspase-activation recruitment domain (CARD) induces NOD1 signaling upon PGN stimulation and consequently interrelates with receptor-interacting protein 2 (Rip-2) adaptor molecules [11]. It has been clearly defined that a component of PGN D-glutamyl-meso-diaminopimelic acid (iE-DAP) exists mainly in Gram-negative, and rarely in Gram-positive, microorganisms, which are sensed by NOD1 [12]. The interaction between NOD1 and a CARD happens through Rip-2, which is essential for NOD1 signaling [13]. As a respondent to the harmful stimuli in the cytoplasm, NOD1 activates innate immunity [14], and the activation of NOD1 initiates various immune responses in the cells, such as inflammation, cytokine transcription, and apoptosis [15,16].

The most composite and essential biological progression that kills and eliminates aggravated cells through bacterial inhibition during animal growth, cellular homeostasis, and disease is known as apoptosis [17,18]. It has been revealed that many apoptotic pathways are initiated through different modes of actions or receptors prompted from the cell surface [19]. Caspases are thought to be the chief mediators of apoptosis [20], and their involvement is generally comprised of two categories: initiators (Caspase-8 and Caspase-9) and executioners (Caspase-3) [21]. Death protease initiation mostly occurs through Caspase-3 by a number of cellular proteins being cleaved and catalyzed [22]. However, many fundamental questions have frequently been raised on Caspase-3 activation in different cell types, for example, whether this protease is essential for cell death or just for the association with apoptosis morphology [19]. Furthermore, important progress has been made in understanding the role of NOD1 regulation during inflammation and autophagy in response to invasive microbes [23,24]. Therefore, we have proposed that an HC diet would induce the apoptosis of mammary epithelial cells by initiation of the NOD1-dependent Caspase-8 pathway through iE-DAP in the mammary gland tissue of dairy cows. However, the actual mechanism and cytosolic localization where NOD1 interrelates with iE-DAP and recruits the adaptor molecule Rip-2 to enable apoptotic responses are poorly understood. Our study was mainly focused on investigating the effects of an HC diet in inducing the apoptosis of mammary gland epithelial cells and the underlying mechanism involved in activation of the NOD1/Caspase-8 pathway via iE-DAP in dairy cows during SARA. 

## 2. Materials and Methods 

### 2.1. Ethics Statement

Nanjing Agricultural University Ethics Committee for animals permitted the design of experiments and sampling procedures before the trial was conducted. The research was performed according to reputed protocols for ‘‘Experimental Animals’’ given by the Ministry of Science and Technology (2006, Beijing, China).

### 2.2. Animals, Diets, and Their Trail Plan

Detailed information about the ingredients and nutrient composition of diets fed to dairy cows was presented in the article by Wang et al. [5].

### 2.3. Collection of Samples

Sterile 5 mL syringe-capped one-layer gauze was used to collect rumen fluid after morning feeding, through a rumen fistula, for three consecutive days during the 18th week, at 0 h interims beginning at 0.5 h for 12 h. The four layers of cheesecloth were used for the filtration of rumen fluid samples. The jugular vein was selected for the collection of blood samples in 5 mL vacuum tubes containing an anticoagulant (sodium heparin). The plasma was isolated from the blood samples by centrifugation at 3000×g at 4 °C for 15 min, and plasma samples were then kept at −20 °C. Slaughtering of all the cows was performed at the completion of the experimental time, and mammary gland tissues were dissected and rapidly kept in liquid nitrogen and later preserved in a −70 °C refrigerator.

### 2.4. Detection of Apoptosis by the Tunnel Assay

To determine the number of apoptotic cells in both groups, we performed a tunnel assay. The paraffin sections of cow tissue samples were fixed on cell climbing slides and dried slightly. The objective area was marked with a liquid blocker pen (GT1001, Gene tech, Shanghai, China) and 50–100 μL of the permeabilizing working solution (G1204, Servicebio technology, Wuhan, China) was added and incubated for 20 min at room temperature. Slides were washed three times with phosphate buffered saline (PBS) solution for 5 min each. Reagent 1 terminal deoxynucleotidyl transferase (TdT) and reagent 2; 2’-deoxyuridine 5’-triphosphate (dUTP) were mixed at the ratio of 1:9 (both from the “Tunnel assay kit Roche 11684817910”). Washing was repeated three times with PBS (pH 7.4) in a Rocker device (TSY-B, Servicebio technology, Wuhan, China), for 5 min each. Then, the samples were incubated with 4′,6-diamidino-2-phenylindole (DAPI) solution at room temperature for 10 minutes in a dark place. The washing step was repeated thrice with PBS (pH 7.4). Each section was mounted on a glass microscope slide with an anti-fad mounting medium (G1401, Servicebio technology, Wuhan, China). All images were detected using Ortho-Fluorescent Microscopy (Eclipse C1, Nikon, Tokyo, Japan) with an imaging system (DS-U3, Nikon, Tokyo, Japan). DAPI radiances in blue were produced by a UV excitation wavelength of 330–380 nm and emission wavelength of 420 nm, whilst fluorescein isothiyocyanate (FITC) radiances in green were produced by an excitation wavelength of 465–495 nm and emission wavelength of 515–555 nm. The Image Pro software was used to determine the number of apoptotic cells.

### 2.5. Isolation of RNA, cDNA Synthesis, and Real-Time (RT-qPCR)

A total of 50 mg powdered mammary gland tissue was taken to isolate the RNA through Trizol (cat. 9108, Takara, Dalian, China), as defined by the manufacturer’s procedure. The spectrometry Eppendorf Bio photometer Plus (Eppendorf AG, Hamburg, Germany) was operated to detect the concentration and quality of total RNA at A260/A280. Samples with 1.8 and 2.1 values were utilized in this experiment. A total of 250 ng/µL of the total RNA template with Takara Co., Otsu, Japan Prime Script RT Master Mix Perfect Real Time was used to synthesize the first-strand cDNA by following the manufacturer’s protocols. qPCR was performed to evaluate the expression of particular mRNA components. The specific primer sequences for NOD1, Rip-2, Caspase-3, Caspase-8, Caspase-9, Bcl-2, and Bax, and β-actin as a housekeeping gene, were designed through primer premier software 5.0 (Premier Bio soft, California, CA, USA). The sequences of used primers are mentioned in Table 1. PCR conditions consisted of 2 µL cDNA and 0.4 µM primers for each reverse and forward primer in 20 µL of the total super mix. Parameters of thermal cycling were as follows: 95 °C for 15 s initial denaturation, 40 annealing cycles at 95 °C for 5 s, followed by 60 °C heating for 31 s primer extension. All the procedures were conducted in triplicate. Amplification of each cDNA was done through SYBR Green (Takara Co., Otsu, Japan) by using the ABI 7300 Fast Real-time PCR System (Applied Bio system, city, USA). The mean of the β-actin housekeeping gene was used to control the irregularity in gene expression levels, and all the data were evaluated through R = 2^-ΔΔCt. Gel electrophoresis in 1.5% agarose was performed for confirmation of the PCR products.

### 2.6. The Concentration of Caspase-3 and Caspase-8 in Mammary Tissue

Mammary gland tissue of dairy cows weighed up to 100 mg was placed in a 2 mL Eppi tube and 1 mL ice-cold radio immune precipitation assay (RIPA) lysis buffer was added (cat. SN338; Sunshine Biotechnology Co., Ltd; Shanghai, China). After that, samples were homogenized through a Dounce homogenizer (Polytron PT 1200 E, Lucerne, Switzerland) and incubated on ice for 30 min, and centrifugation of the sample was done at 12,000 rpm for 15 min at 4 °C to collect the supernatant. The Protein Assay Kit named Pierce TM BCA (cat. 23225, Thermo Fisher, Waltham, MA, USA) was utilized to detect the concentration of protein. All the protein samples were diluted according to the same final level. Caspase-3 and Caspase-8 activation was determined by an enzyme activity assay kit, according to the manufacturer’s protocol (KeyGEN Biotech. Co. Ltd, Nanjing, China), as defined earlier [25].

### 2.7. Western Blotting Analysis

The method employed for total protein separation has already been mentioned in the overhead section. After that, the protein concentration was measured with the bicinchoninic acid (BCA) protein assay kit (Pierce, Rockford, IL, USA). The proteins were loaded in equal amounts for separation using 10% SDS-polyacrylamide gel electrophoresis (PAGE), and a nitrocellulose (NC) membrane (Biosharp, China) was then used to transfer the proteins at 4 °C. Either 10% skim milk or 10% bovine serum albumin (BSA) “for phosphorylated proteins detection” was used for blocking of the membrane with a Tween (TBST) buffer at room temperature for 2 h. Afterward, the NC membranes were washed thrice with 1 TBST for 10 min each and incubated with specific primary antibodies (NOD1, Caspase-3, Caspase-3 cleaved, Caspase-8, Bax, and Bcl-2) at 4 °C overnight (1:1000 dilution, antibody: TBST Beyotime, Shanghai, China), followed by washing and incubation of the membranes with the corresponding secondary antibodies for 2 h at room temperature. As a final step, an enhanced chemiluminescence detecting kit (ECL) (Vazyme Biotech. Co., Ltd; Nanjing, China) was used to treat the membrane and record the signals by the LAS4000 imaging system (GE Healthcare Biosciences AB, Uppsala, Sweden). For all the antibodies used in this experiment, their details were taken from previously published papers [23,25,26]. The results were quantified through the Image Lab software 5.2 versions (Bio-Rad, California, CA, USA).

### 2.8. Immunofluorescence Antibody (IFA) Assay

To further confirm the results of western blotting, we performed immunofluorescence microscopic analysis. The paraffin sections of tissue samples from dairy cows were incubated twice with xylene (SCRC), for 15 min each. Slides were fixed in absolute ethanol (SCRC) and dried with 85% and 75% gradient ethanol for 5 min each. They were then washed with distilled water and dipped in Ethylene di amine tetra acetic acid (EDTA) antigen retrieval buffer (pH 8.0) (G1206, Servicebio technology, Wuhan, China). The slides were maintained at a sub-boiling temperature for 8 min, followed by another sub-boiling temperature for 7 min. They were washed thrice with PBS (pH 7.4) in a Rocker device (TSY-B, Servicebio technology, Wuhan, China) for 5 min each. Antigen retrieval buffer was used and heated according to the sample tissue characteristics. The deceptive liquid was aspirated and objective tissue was marked with the liquid blocker pen (GT1001, Gene tech, Shanghai, China). Fluorescence quenching reagent (G1221, Servicebio technology, Wuhan, China) was added on the slides and incubated for 5 min, and then washed with running tap water. Next, the slides were incubated with a specific primary antibody of NOD1 and Caspase-8 (same as used for western blotting) diluted in a “1:100” ratio overnight at 4 °C, and placed in a wet box containing water. Slides were washed three times with PBS (pH 7.4) in a Rocker device for 5 min each and then the slides were dried carefully underneath and around the sample. All the slides were incubated with a respective secondary antibody (the same used for western blotting) for 1 h at 4 °C in the dark. Then slides were washed three times with PBS (pH 7.4) in a Rocker device for 5 min each and incubated with DAPI (G1401, Servicebio technology, Wuhan, China) for 10 min at room temperature in the dark. Subsequently, three drops of glycerol (anti-fade mounting medium G1401, Servicebio technology, Wuhan, China) were added and a coverslip was placed by avoiding the formation of air bubbles. Finally, slides were observed under Fluorescent Microscopy (Eclipse C1, Nikon, Tokyo, Japan) with an imaging system (DS-U3, Nikon, Tokyo, Japan). DAPI emitted blue fluorescence by UV excitation at 330–380 nm wavelengths and the emission wavelength was 420 nm. The Cyanine (CY3) emitted red fluorescence by excitation at 510–560 nm wavelengths and the emission wavelength was 590 nm. Photographs were taken of every section of the sample.

### 2.9. Statistical Analysis

Evaluation of the data was done through SPSS software version 17.0 (SPSS Inc., Chicago, IL, USA), all data remained publicized as the mean ± standard deviation (SD), and alterations among the groups were evaluated by the Student t-test and one way ANOVA to assess pH data. When *p* < 0.05, the variances were significant. The graphs were plotted with the Graph pad prism 07software (GraphPad Instat Software, California, CA, USA).

## 3. Results

### 3.1. Determination of Apoptotic Cells in the Mammary Gland Tissue of Dairy Cows

**Figure 1:**
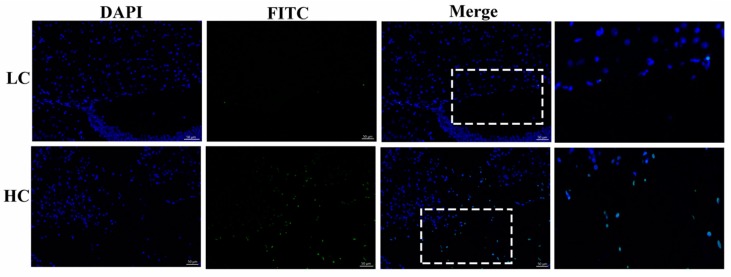
Determination of apoptotic cells by the tunnel assay. The HC (high-concentrate) group showed a significant amount of positive apoptotic cells (white arrows) in contrast to the LC (low-concentrate) group. The nucleus was made blue by labeling with DAPI and the positive apoptosis cells are green (FITC), white rectangular selected area shows an enlarged area. The images were taken at a 50 µm scale bar.

The HC group exhibited a significant number of positive apoptotic cells in comparison to the LC group in the mammary gland tissue of dairy cows. The number of apoptotic cells in the HC group (139 ± 30.89) was higher than in the LC group (9.33 ± 0.882), as shown in Figure 1. The results were obtained in triplicate.

### 3.2. mRNA Expression of NOD1, Rip-2, Bax, and Bcl2 in Mammary Gland Tissue

**Figure 2:**
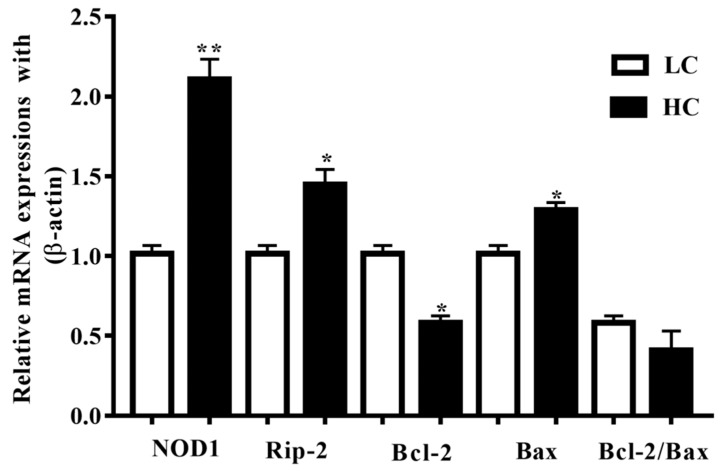
Relative mRNA expressions of NOD1, Rip-2, Bax, and Bcl-2 in comparison with the β-actin universal control. NOD1 and Rip-2, and Bax mRNA expressions in the mammary gland tissue were higher in the HC group than in the LC group. Conversely, the Bcl-2 mRNA expressions were markedly lower in the HC group than in the LC group. * *p* < 0.05 and ** *p* < 0.01 versus control.

NOD1 and Rip-2 mRNA expressions in the mammary gland tissue were higher (*p* < 0.01) in the HC group in contrast to the LC group (*p* < 0.05). Additionally, Bax mRNA expressions in the HC group were notably higher (*p* < 0.04) than in the LC group, but the Bcl-2 mRNA expressions were markedly lower (*p* < 0.02) in the HC group than in the LC group (Figure 2).

### 3.3. Determination of Caspase-3, Caspase-8, and Caspase-9 mRNA Expressions, and Caspase-3 and Caspase-8 Concentration in Mammary Gland Tissue

**Figure 3:**
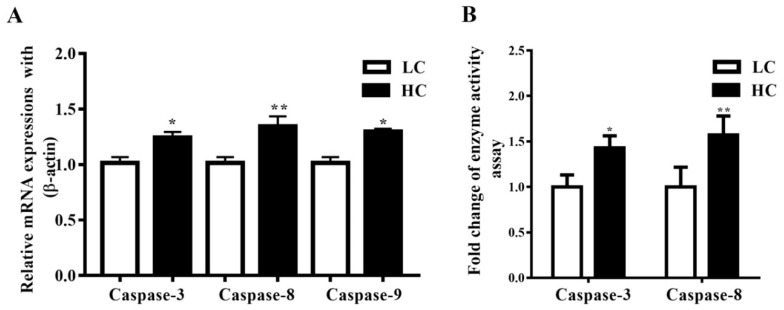
(**A**) In the mammary gland tissue, the mRNA expression of Caspase-3, Caspase-8, and Caspase-9 was markedly increased in the HC group compared to the LC group. (**B**) The concentration of Caspase-3 was markedly higher in the HC group than in the LC group. However similar mRNA trends were exhibited by Caspase-8. * *p* < 0.05 and ** *p* < 0.01 versus control.

Caspase-3, Caspase-8, and Caspase-9 mRNA expressions in the mammary gland tissue of dairy cows were observed to be markedly higher (*p* < 0.04, *p* < 0.01, and *p* < 0.05) in the HC group compared to the LC group (Figure 3A). Moreover, the activation of Caspase-3 was markedly higher (*p* < 0.05) in the HC group than in the LC group. Conversely, parallel to the LC diet, the HC diet increased the activation of Caspase-8 (*p* < 0.01), as shown in Figure 3B.

### 3.4. Protein Expression in Mammary Tissue

**Figure 4:**
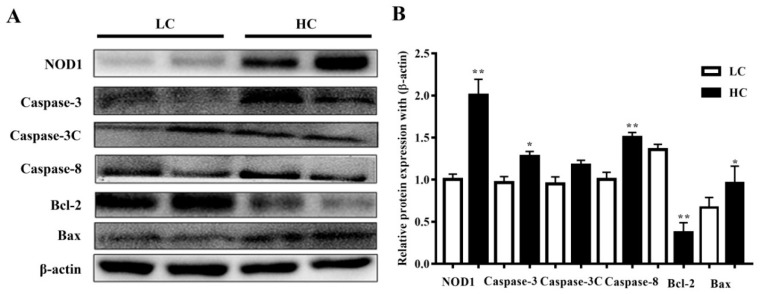
(**A**) The protein expressions of NOD1, Caspase-3, Caspase-8, and Bax were observed to be higher in the mammary gland tissue of the HC group compared to the LC group. Bcl-2 protein expressions were markedly higher in the LC than in the HC group. (**B**) Figure b represents their histogram. * *p* < 0.05 and ** *p* < 0.01 versus control.

NOD1 (108 kDa) and Caspase-3 (35 kDa) protein expressions, and Caspase-8 (48 kDa) and Bax (21 kDa) expressions in the HC group in the mammary gland tissue of dairy cows were markedly higher than in the LC group. However, the Bcl-2 (28 kDa) protein expressions were considered significant in the LC group in comparison to the HC group. However, there were no substantial differences observed in the activation of cleaved Caspase-3 (17 kDa) in both groups. The data are presented in Figure 4.

### 3.5. Protein Expression and Localization of NOD1 and Caspase-8

**Figure 5:**
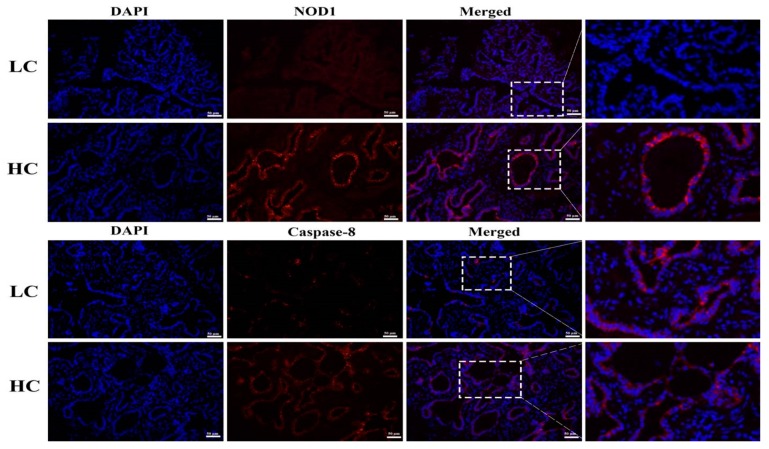
Localization of NOD1 and Caspase-8 proteins was detected through IFA. An increased level of red staining (CY3) of NOD1 and Caspase-8 proteins was observed in the HC group compared to the LC group in mammary tissue epithelial cells of dairy cows, with DAPI blue nucleus staining being conducted during the immunofluorescence examination. The images were taken at a 50 µm scale bar. The white rectangular selected area shows an enlarged area.

To further confirm the western blotting results, we performed IFA. The HC group showed significant increases in NOD1 protein localization in the mammary tissue epithelial cells of dairy cows than the LC group. Similarly, increased staining of the Caspase-8 in HC groups was observed in comparison to the LC group. The nucleus is labeled with DAPI blue, whilst specific antibodies for NOD1 and Caspase-8 localization are marked with CY3 red, as shown in Figure 5.

## 4. Discussion

Due to higher demands, dairy ruminants are raised with an HC diet to increase milk production. However, these diets can only increase the quality and milk yield in the short term, whereas long-term use of an HC diet leads to a series of adversities in dairy ruminants, including decreased rumen pH values, iE-DAP production and translocation, gastrointestinal barrier disruption, and multiple diseases, such as SARA [27]. Previous studies have suggested that the experimental induction of SARA is generated upon grain-based challenges [1,28,29]. The most reliable and precise approach for diagnosing SARA is the detection of pH in rumen fluids [30]. Wang et al. proved that the ruminal pH values were all witnessed to be below 5.8 in the HC group for a continuous 3 h period, signifying that SARA was proficiently incited [5], and the results from our recently published article are consistent with those of previous studies [2,31]. According to previous reports, high-concentrate feeding enhances the amount of ruminal iE-DAP [32], which is usually found in Gram-negative and some Gram-positive bacteria in the rumen [33]. When the ruminal pH is depressed, the amount of iE-DAP can be increased and translocated into the bloodstream [34,35]. Wang, et al. revealed that due to pH depression, the concentration of iE-DAP detected in the rumen liquor of the HC group (9.82 μg/mL) was markedly elevated in comparison to that in the LC group (5.45 μg/mL), and translocated iE-DAP can ultimately stimulate innate immunity in dairy cows [5]. 

The most complex and crucial biological progression that destroys and eliminates infuriating cells through the inhibition of microbes during animal growth, cellular homeostasis, and disease is a well-known process called apoptosis [17,18]. Previous studies have been widely conducted to examine and understand the apoptotic pathways and their protein mechanisms [36]. However, the NOD1 apoptotic pathway is still not well-characterized for mammary gland tissue of dairy cows, which is thought to be regulated by NOD1 upon microbial sensing. The caspases are divided into initiators and executioners, and the initiator caspases are the first to be activated during apoptosis [20]. Precisely, we studied NOD1-dependent Caspase-8 pathway activation and Bcl-2 regulation to confer the cell apoptosis induced by an HC diet via the translocation of iE-DAP in the mammary gland of dairy cows. Here, we found that the transcriptional regulation of NOD1, Rip-2, Caspase-3, Caspase-8, and Caspase-9 showed significantly higher expression in the HC group in comparison to the LC group, determined through RT-qPCR. Additionally, the protein expression of NOD1, Caspase-3, and Caspase-8 was also up-regulated in the HC group, as detected through western blot analysis. As reported earlier, NOD1 can control the progression of cellular apoptosis by stimulating apoptosis-associated gene expressions, such as Caspase-8, a key initiator in NOD1-dependent apoptosis, and Rip-2 consequently engages with NOD1 by CARD-CARD interfaces and generates the proceedings that lead to cell death through Caspase-8 [37]. During an apoptotic process, the death receptors initiated and bound to their specific receptors by the mitochondrial apoptotic pathway, and Caspase-9 or Caspase-8 initiation can cleave and activate Caspase-3, leading to apoptosis straight away [38]. As described previously, cleavage is neither sufficient nor required to confirm the activation of initiator caspases [39]. To examine the downstream events of apoptosis, we detected the concentration of Caspase-3 and Caspase-8 and found that both were markedly higher in the HC group than the control group. In contrast to the present study, programmed cell death is vitally mediated by the caspase family; for example, a commonly triggered death protease is Caspase-3 and its vital contribution takes place during cell death in various, but not all, circumstances, such as in particular cell types, tissues, or stimuli of death, in a significant manner [40]. However, the regular initiation of Caspase-3 in various types of cells has stimulated some critical interrogation into whether Caspase-3 is required for morphological variations or correlated to cellular apoptosis [19,22], so the presence of other Caspase-3-like proteases should not be ignored. Immuno-labeling further confirmed the localization of NOD1 and Caspase-8 in mammary gland tissue epithelial cells, and we found more positive CY3 staining in the HC group, which showed a similar trend to that observed in the case of western blot analysis.

Bcl-2 is one of the crucial anti-apoptotic proteins and it has been reported to have a key influence in extended cell survival by preventing apoptosis [41], which was up-regulated in the LC group in comparison to in the HC group. Generally, in a vigorous cell, Bcl-2 stays bound to one of the pro-apoptotic associates, for example, Bax, until activation of the apoptosis stimulus takes place, and Bax is the protein that helps in the formation of pores in the mitochondrial membrane within the cells via homo di/oligomerization [42]. Additionally, in the HC group of dairy cows, both the mRNA and protein expression of Bax was significantly increased, while Bcl-2 mRNA and protein expression were significantly decreased. The Bcl-2 to Bax ratio is mostly used to reveal the anti-apoptotic ability of cells [43]. Based on our findings, we confirmed that nourishing the dairy cows with an HC diet for up to 20 weeks can efficiently incite SARA by decreasing the rumen pH for up to 3 hours/day, which eventually increases the iE-DAP concentration that, on translocation, prompts mammary epithelial cell apoptosis by activating the NOD1/Caspase-8 pathway. Moreover, in vivo and in vitro studies are required to understand and magnify the emerging role of NOD1 in the induction of cellular apoptosis. 

## 5. Conclusions

Dairy cows fed with an HC diet can efficiently provoke SARA by decreasing the ruminal pH and increasing the amount of iE-DAP. Importantly, we found that iE-DAP translocation into the bloodstream induces mammary epithelial cell apoptosis by triggering the NOD1/Caspase-8 pathway in mammary glands of dairy cows. Our findings indicate a new era of the NOD1-dependent Caspase-8 pathway in inducing mammary epithelial cell apoptosis.

## Figures and Tables

**Table 1:** List of primers and their sequence used for qRT-PCR in dairy cows.

Gene	Accession Number	Primer Sequence (5′–3′)		Product Length (bp)
NOD1	NM_001256563.1	Forward	TCAACACTGACCCAGTGAGC	147
		Reverse	TGAAGTTGACCAGCTCCACC	
β-actin	AY141970	Forward	CTCTTCCAGCCTTCCTTCCT	178
		Reverse	GGGCAGTGATCTCTTTCTGC	
Rip-2	NM_001034610.2	Forward	ATTCTGGTCCACGGGAGGAGTC	94
		Reverse	TCTTGAGGAGCTGACAGGGACC	
Caspase-3	NM_001077840.1	Forward	CAGCGTCGTAGCTGAACGTAA	227
		Reverse	ATCGACAGGCCATGCCAGTAT	
Caspase-8	NM_001045970.2	Forward	AGCAAATGGTCCAGGCTTTG	250
		Reverse	GCTCTTGTTGACCTGCTCAC	
Caspase-9	NM_001205504.1	Forward	AGCAAATGGTCCAGGCTTTG	160
		Reverse	ATTCTCTCGACGGACACAGG	
Bcl-2	NM_001075417-2	Forward	AGGTTGGTAACCGGACCCTA	174
		Reverse	TTCCTGCCTGTCCTCGAATG	
Bax	NM_173894.1	Forward	GCTGTGGACACAGACTCTC	158
		Reverse	CTGATCAACTGGGCACCTT	
